# Release of Tretinoin Solubilized in Microemulsion from Carbopol and Xanthan Gel: In Vitro versus Ex Vivo Permeation Study

**DOI:** 10.3390/polym15020329

**Published:** 2023-01-09

**Authors:** Miroslava Špaglová, Martina Papadakos, Mária Čuchorová, Desana Matušová

**Affiliations:** Department of Galenic Pharmacy, Faculty of Pharmacy, Comenius University in Bratislava, Odbojárov 10, SK-832 32 Bratislava, Slovakia

**Keywords:** tretinoin, lecithin, microemulsion, carbopol, xanthan gum

## Abstract

Background: Tretinoin (TRE) is, for its anti-comedogenic and comedolytic activity, widely used in the topical treatment of acne vulgaris. The effect lies in the regulation of sebum production and collagen synthesis. The study is devoted to the formulation of dermal gels containing TRE using microemulsion as the drug solubilizer. Methods: The aim was to evaluate the effect of the reference microemulsion (ME) and lecithin-containing microemulsion (ME_L_) on the release of TRE through the synthetic membrane (in vitro) and the pig’s ear skin (ex vivo) through the Franz cell diffusion method. Subsequently, after an ex vivo study, the amount of the drug in the skin influenced by the applied formulation was determined. In addition, the impact of ME on the microscopic structure, texture, and rheological properties of gels was evaluated. Results: On the basis of the analysis of texture, rheological properties, and drug release studies, Carbopol formulations appear to be more appropriate and stable. Considering the synthetic membrane as a *stratum corneum*, the Carbopol gel penetrated about 2.5-higher amounts of TRE compared to the Xanthan gel. In turn, ex vivo studies suggest that ME_L_ slows the drug transfer to the dissolution medium, simulating absorption into the blood, which is a desirable effect in local treatment. The drug retention study proved the highest amounts of TRE in the skin to which microemulsion-Carbopol formulations were applied. Conclusion: The results confirm the benefit of TRE solubilization in ME due to its bioavailability from the tested dermal formulations.

## 1. Introduction

Tretinoin (TRE), synonym all-*trans*-retinoic acid, (chemically (2E,4E,6E,8E)-3,7-dimethyl-9-(2,6,6-trimethylcyclohexen-1-yl) nona-2,4,6,8-tetraenoic acid) [1], belongs to the first generation of retinoids. It is widely used for the topical treatment of various skin disorders, such as acne vulgaris and skin aging caused by UV radiation (photo-aging) [2]. It may also be used in so-called off-label indications, such as psoriasis, ichthyosis, follicular keratosis, stretch marks, verruca vulgaris, cutaneous warts [3], cutaneous lupus erythematosus [4], melasma (hyperpigmentation) [5], and squamous cell carcinoma [6]. Beyond that, it seems to be helpful in the prevention of non-melanoma skin cancer [5]. Oral administration of TRE is approved by the FDA (U.S. Food and Drug Administration) for the treatment of acute promyelocytic leukemia, moderate to severe acne, and cystic acne [3].

It was applied in dermatological therapy for the first time in 1959 [7]. Due to the severe side effects of systemic treatment, TRE is administered almost exclusively topically [8]. The only clinical indications for the oral administration of TRE approved by the FDA (U.S. Food and Drug Administration) are acute promyelocytic leukemia and moderate to severe cystic acne [3]. TRE similarity to isotretinoin (a recognized human 13-*cis* retinoic acid) has caused concerns about the teratogenic potential of topically applied TRE [9]. Although retinoid embryopathy was found after oral administration, there is still insufficient knowledge about it after topical treatment [10]. Melhorn [11] in turn stated that malformation in newborns was reported also after topical application.

Acne vulgaris is a common chronic skin disease caused by the obstruction and/or inflammation of pilosebaceous units such as hair follicles and their associated sebaceous glands [12]. It is developed as a consequence of several processes: hypercornification, plugging of the follicular opening by sloughed keratinocytes and other debris, comedone formation, excess sebum production, abnormal proliferation of *Propionibacterium acnes*, and leukocyte infiltration [13]. The mechanism of TRE action is binding to specific nuclear receptors, and the subsequent interference with gene expression requires proliferation, differentiation, sebum production, inflammation, and immunological reactions.

Topical TRE application may lead to local skin irritation. The limited TRE stability in oxygen, light, and acidic pH leads to efficacy loss during storage [8]. Its low water solubility limits incorporation into hydrophilic vehicles [14]. Furthermore, available topical formulations such as creams, gels, lotions, emulsions, and others allow only limited efficacy of the drug due to the skin barrier properties and the relatively low TRE stability in them [8]. The photo-degradation of retinoic acid is a complex problem involving several degradation processes, the most important of which is isomerization. TRE underlies isomerization by radiation to 13-*cis* retinoic acid (isotretinoin) and further to 9-*cis* retinoic acid [15]. Ourique et al. [14] found out that after 8 h of TRE exposure to UV radiation, 68.64 ± 2.92% of the drug undergoes degradation. To prevent the decomposition by light and achieve the desired effect, the application of TRE products is recommended in the evening [16]. TRE properties limit its topical application, namely poor water solubility, high chemical instability, photo-instability, and irritant potential [17]. Drug carriers allow the administration of retinoids in an aqueous vehicle. TRE encapsulation in a nanocarrier leads to an improvement in drug stability, drug delivery, and thus an increase in the effectiveness of skin disease treatment [4,8,17]. Therefore, the selection of a suitable one is important.

Because the topical application of TRE is associated with the mentioned difficulties, the development of a modern formulation with drug nanocarriers is a challenge. Several studies have investigated the stability and bioavailability of TRE encapsulated in different colloidal drug delivery systems [18]. In the present study, the function of the microemulsion as the drug solubilizer and penetration enhancer for the improvement of TRE bioavailability was examined. Based on previous results, where minoxidil was used as the model of a poorly water-soluble topically applied drug [19], it was assumed that the lecithin-containing microemulsion could significantly affect the residual amount of TRE in the skin and prevent its delivery into the bloodstream. Although many colloidal delivery systems for improving TRE bioavailability and stability after dermal application have been designed, true microemulsion systems, as one of the most available remedies, have not. Compared to others, they have one huge advantage—simple preparation without the need for special equipment—and they form spontaneously, which ultimately explains their low price. In our study, TRE solubilized in the microemulsion was finally dispersed in the gel. Hydrogels with their cross-linked three-dimensional network filled with high water content [20] represent a suitable vehicle for dermal application.

## 2. Materials and Methods

Tretinoin (TRE; purity 99%; mol. weight 300.4) and Xanthan gum were purchased from Sigma Aldrich (Darmstadt, Germany). Ethanol (96%), sodium hydroxide, isopropyl alcohol, isopropyl myristate, and Tween^®^ 80 were purchased from CentralChem (Bratislava, Slovakia). Carbopol^®^ 940 was from S&D Pharma (Praha, Czech Republic). Gelatin was purchased from Valuch spol. s r.o. (Banská Bystrica, Slovakia). Propylene glycol was from Interpharm (Bratislava, Slovakia). Soybean lecithin was from Dimica (ASP, Divina, Slovakia). The distilled water used for all experiments was prepared by the distillation apparatus Kavalier (Merci, Brno, Czech Republic) directly at the Department of Galenic Pharmacy (Faculty of Pharmacy, Bratislava, Slovakia).

### 2.1. Solubility of Tretinoin

TRE solubility in the individual hydrophilic components of the microemulsions (water, isopropyl alcohol, Tween 80) and the microemulsions themselves was determined through the solubility equilibrium test according to the modified method [21], similar to [19]. TRE (1%; *w*/*w*) was dissolved in the solvent or the ME for 50 h during continuous stirring on an electromagnetic stirrer at laboratory temperature. Subsequently, the supernatant was separated from the insoluble sediment by centrifugation at 3000 rpm (CompactStar CS4, VWRInternational GmbH, Vienna, Austria). The supernatant was filtered through a syringe filter (Q-Max^®^ RR, 0.45 µm, Lambda Life, Bratislava, Slovakia). The samples were diluted with a binary solution of ethanol (96%) and distilled water (1:1). The drug concentration was determined by a spectrophotometer (Genesys 10 UV-VIS, Cambridge, UK) at 337 nm. The drug concentration was calculated using the equation of the standard curve describing the linear regression, concentration *c* versus absorbance *A*, taking the dilutions of the samples into consideration.

### 2.2. Preparation of Microemulsions

The microemulsions were prepared by the so-called “titration method”. The surfactant, the co-surfactant, and the oil phase were mixed on a magnetic stirrer until a clear solution appeared, and subsequently water was added drop-by-drop until a clear microemulsion arose. During ME_L_ production, lecithin was first dissolved in the oil phase. The complete composition is reported in Table 1. A more detailed characterization of the microemulsions is described in a previous study [19].

### 2.3. Preparation of Gels

Two gels—Carbopol and Xanthan—were used in the experiment, and they were prepared as follows: The gelling agent (Carbopol or Xanthan gum) was left to swell in half of the total water amount for 30 min. Subsequently, the required amount of purified water was replenished so that the final dispersions consisted of 1% (*w*/*w*) of Carbopol and 1.5% (*w*/*w*) of Xanthan gum. The dispersions were continuously stirred until a homogenous gel structure was formed. Moreover, in the Carbopol gel, just before the addition of the total amount of purified water, the sodium hydroxide solution (10%, *w*/*w*) was dropped gently to neutralize the dispersion to pH 7.

### 2.4. Characterization of Gels

Gels were characterized in terms of their rheological behavior by viscometer Rheoloab QC (Anton Paar, Graz, Austria). Farrow’s constant was found by plotting the logarithm of shear stress versus the logarithm of shear rate. The influence of ME on the gel texture was analyzed by the texture analyzer TA.XT Plus (Stable Micro Systems Ltd., Godalming, UK). The vial was filled with an equal volume of gel or ME + gel (15 mL). The vials were placed in an ultrasonic water bath for 15 min to remove air bubbles. Texture analysis conditions: test-compression, pre-test speed: 5.00 mm/s, test speed: 2.00 mm/s, post-test speed: 5.00 mm/s. The probe recorded a force at the moment of passing through the surface of the sample, and at that moment it slowed down to a speed of 2 mm/s. The analyzed sample distance was 5 mm. The analysis was performed in two cycles without interruption. The data were evaluated using the software Texture Exponent 32 (3.0.5.0) and subsequently, due to the dependence of the force corresponding to the resistance of the sample over time, the texture properties of the samples such as strength, cohesion, and adhesion were expressed.

The microstructure of the ME-containing gels was observed by microscope LEICA DM E (Leica Microsystems GmbH, Wetzlar, Germany).

### 2.5. Preparation of Tretinoin Gels

The amount of TRE (2%; *w*/*w*) in the gel formulation was solubilized in the tested microemulsion formulations (ME or ME_L_) or dissolved in a mixture of ethanol (96%; *w*/*w*) and propylene glycol (1:1). The solvent mixture was used as the reference solubilizer in the comparative formulations. Brisaert and Plaizier-Vercammen [22] have stated that TRE is an effective comedolytic agent of the highest activity when solubilized in the mentioned solvents at the same wt. %. The dissolution was accelerated by stirring on an electromagnetic mixer (Magnetic Stirrer type MMS-3000, Biosan SIA, Latvia). Then, the appropriate amount of gel vehicle was added so that the final ME/or solvent to gel ratio was 1:4 followed by stirring until homogeneous appearance.

TRE undergoes isomerization under light [23] and irradiation of its aqueous solution results in the formation of up to nine different isomers, from which isotretinoin is predominant [24]. Raza et al. [2] reported that 70% of TRE was degraded after 10 min of irradiation. Photo-instability of this substance is a problem in the development of a suitable topical product. To minimize the risk of drug degradation, all experiments were performed under special conditions, in a darkened room. 

### 2.6. In Vitro Drug Release

The in vitro drug release was determined using vertical Franz cells (J&J glass, Bratislava, Slovakia). A semi-permeable membrane (Spectra/Por^®^ 4 dialysis membrane, Spectrum Laboratories, Inc., U.S.) was attached to the donor apparatus compartment. The diffusion area was 2.34 cm^2^ ± 0.07. The acceptor chambers (volume 28.5 mL ± 0.57) were filled with a dissolution medium, water-ethanol (96%) (1:1), permanently heated to 32 ± 0.5 °C [2], simulating the physiological skin temperature [14]. The continuous electromagnetic stirring of the dissolution medium was ensured during the whole time of the drug release. The sample (0.6 g) was applied to a semi-permeable membrane of the donor compartment fixed on acceptor chambers with the dissolution medium. At a one-hour time interval of six hours, the equal volume (4 mL) of the acceptor solution was withdrawn and immediately replenished with the dissolution medium. For each gel formulation, six parallel measurements were performed, and values were expressed as a mean ± S.D. The concentration of TRE released was then determined spectrophotometrically at 337 nm.

The standard curve equation was found to be the following: y = 0.1208 x − 0.009 (with a correlation coefficient R^2^ = 0.9998) from which the concentration of the released drug *c* (µg·mL^−1^) was calculated. The cumulative drug concentration *Q_t_* was calculated according to the Equation (1):

(1) $$Q_{t}=V\;C_{t}+\overset{t-1}{\underset{i=0}{\sum }}V_{1}\;C_{i}$$

where *C_t_* is the drug concentration in the acceptor compartment at sampling time *t* (µg·mL^−1^), *C_i_* is the drug concentration of the *i*-th sample (µg·mL^−1^), *V* is the volume of the acceptor compartment (≈28.5 mL), and *V*_1_ is the volume of the sample taken (5 mL). The cumulative percentage *X* (%) of the released drug was calculated according to the Equation (2):

(2) $$X=\frac{Q_{t}}{Q_{total}}\;100$$

where *Q_total_* is the total amount of tretinoin (µg) in gel formulation released per unit membrane/skin area [25,26].

### 2.7. Ex Vivo Drug Permeation Study

The ex vivo drug permeation study was performed similarly to the in vitro drug release, with only one difference. The porcine ear skin (Stará Huta, Slovakia) was used instead of the semi-permeable membrane. This animal model of the skin is preferred for ex vivo experiments because of its similarity to human skin in terms of biochemical properties, follicle density, and thickness [24]. The intact and not heat-treated ear skin from six-month-old slaughtered pigs was immediately washed with distilled water (25 ± 2 °C) after removal and stored at −18 ± 2 °C until the ex vivo study was performed (max. of 2 weeks). Then, 12 h before use, they were defrosted at room temperature. After thawing, the porcine auricle skin was separated from the cartilage, and again washed with distilled water and saline. Hair was gently removed by cutting. Intact skin pieces (3 × 3 cm) were selected, cut, and then fastened to the donor chamber in such a way as to arrange the contact of the applied gel sample with the *stratum corneum*. The blind test was performed the same way, without the applied gel sample on the skin. The following procedure, as well as the dissolution medium, were consistent during the in vitro drug release study. The drug concentration was determined spectrophotometric at 337 nm. The results of the blind test were taken into account in the calculation of the drug concentration.

### 2.8. Drug Release Kinetics

The drug release kinetics were determined by the comparison of the coefficients of determination (R^2^) of three conventional kinetics models: zero-order kinetics (data obtained from the drug release studies were plotted as cumulative percentage of drug released versus time), first-order kinetics (data obtained were plotted as log cumulative percentage of drug remaining versus time), Higuchi model (data obtained were plotted as cumulative percentage of drug released versus square root of time) [27].

### 2.9. Drug Deposition in Skin

After six hours of the ex vivo permeation study, the gel was removed from the skin. An unnecessary part of the skin that was not in direct contact with the gel during the permeation study was cut. Thus, the prepared skin pieces were placed in vials with 15 mL of ethanol (96%) and left to macerate at 4 °C for 24 h. Subsequently, the skin pieces were centrifugated (CompactStar CS4, VWR International GmbH, Vienna, Austria) at 3000 rpm for 30 min. The solutions were filtered through a high purity syringe filter (0.45 µm, Q-Max^®^ RR, Lambda Life, Bratislava, Slovakia) and diluted with the ethanolic solution used as the dissolution medium. The drug content (µg/mL) was determined spectrophotometrically.

### 2.10. Statistical Analysis

A data analysis was performed using the software Microsoft^®^ Excel 2016. The results are expressed as the mean ± S.D. To compare the microemulsion-containing gel formulation against the reference gel (without ME), a paired Student’s t-test was applied. Data were analyzed as statistically significant at *p* ˂ 0.05.

## 3. Results

### 3.1. Solubility of Tretinoin

To compare the drug solubility in the individual hydrophilic MEs’ components and MEs themselves, the accelerated test of the drug solubility equilibrium was performed. TRE solubility decreased in the following range: ME_L_ ˃ ME ˃ IPA ˃ Tween^®^ 80 ˃ water. The values are recorded in Table 2.

### 3.2. Preparation and Characterization of Gels

Due to the low viscosity of the microemulsions, they alone were considered unsuitable for topical or transdermal administration [28]. Therefore, TRE was solubilized in the ME and then formulated into the hydrogel. The poor water solubility of TRE is a limiting factor of its incorporation into any aqueous vehicle or bases. Therefore, it requires the use of co-solvents such as ethanol or propylene glycol in commercial products [2]. Many other poorly water-soluble drugs’ potentials in topical administration are difficult to process into any topical dosage form. One of the ways to improve their delivery and thus increase their bioavailability is to present drug carriers such as liposomes, nanoparticles, self-emulsifying systems, nano- and microemulsions, etc. [29].

#### 3.2.1. Optical Microscopy

**Figure 1:**
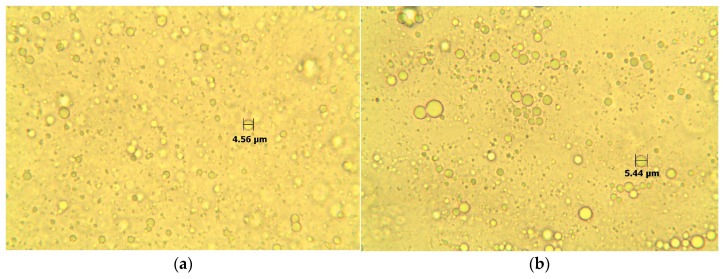
Microscopic gel structure: (**a**) Carbopol gel after adding the reference microemulsion (400× magnification); (**b**) Xanthan gel after the adding of the reference microemulsion, (400× magnification).

The microstructure of Carbopol gel consists of cross-linked spherical polymer particles filled with a dispersing medium (water). After adding the microemulsion, larger droplets belonging to the oil phase of the microemulsion appeared in the structure. This means that after mixing the microemulsion into the gel, the microemulsion broke, and the droplets of its internal dispersed phase increased from nanometers to micrometers. Therefore, they are also observable with an optical microscope. As can be seen, the microstructure of Carbopol (Figure 1a) and Xanthan gel (Figure 1b) differed only slightly. In Xanthan gel, the microemulsion was also broken.

#### 3.2.2. Rheology

Colloidal gels form by aggregating particles, the size of which varies from 10 nm to several micrometers, suspended in the liquid. Their most representative property is the ability to change from a semi-solid state to a liquid under external mechanical stress. Understanding the mechanical responses of colloidal gels, i.e., their rheological behavior, is therefore essential. Depending on the type of polymer and its interconnecting structures, respectively, different gel structures are formed that can react to mechanical stress differently. Generally, two types of gels are distinguished: (a) reversible, the structure of which is restored after the mechanical action has subsided; and (b) irreversible, which has an irreversibly broken structure due to the mechanical stress [30].

**Figure 2:**
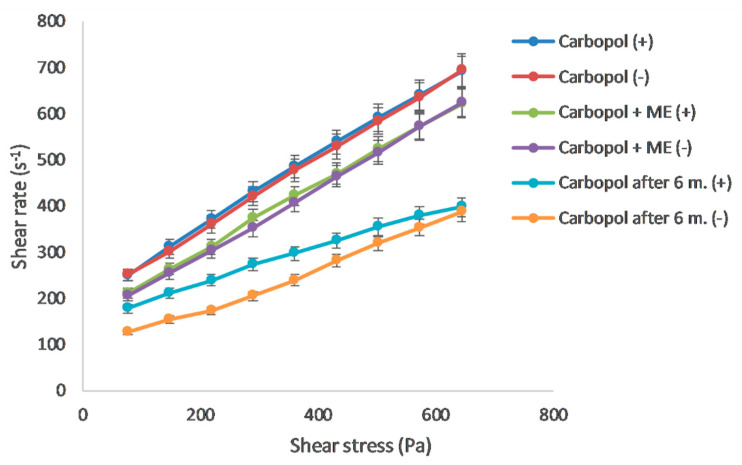
Rheogram for Carbopol gel, ME-containing Carbopol gel, and Carbopol gel after 6 months; (+) up-curve, (−) down-curve.

**Figure 3:**
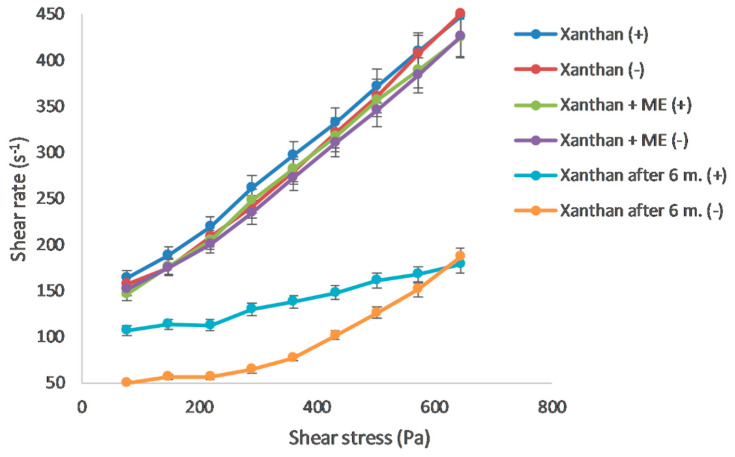
Rheogram for Xanthan gel, ME-containing Xanthan gel, and Xanthan gel after 6 months; (+) up-curve, (−) down-curve.

As the results of the rheological measurement indicate, both Carbopol and Xanthan gels are reversible. Their structure was restored after the stress quit. The rheogram showing the dependence of share stress on share rate (Figure 2 and Figure 3) confirms the pseudoplastic flow of Carbopol gel. The values measured at the increasing and decreasing share rates were almost identical. The addition of microemulsion did not cause significant changes in the rheogram. In contrast, the Xanthan gel rheogram showed the thixotropic properties of the system, manifesting itself as two different curves in the rheogram. The gel structure was disturbed and did not recover until the end of the measurement. During this time, sol was temporarily formed. The presence of microemulsion caused the hysteresis area to slightly increase. The flow characteristics were further analyzed by a regression analysis: the dependence between the logarithm of share stress and the logarithm of share rate with the corresponding Equation (3) [31]:

log D = n log τ − log η(3)

where D represents the share rate (s^−1^), τ share stress (Pa), η viscosity (mPa.s), and n Farrow’s constant, which is a deviation of the flow from the Newtonian one. In the case of pseudoplastic flow, it was higher than 1, for the dilatant flow less than 1. Table 3 shows the corresponding Farrow’s constant values, which, for both gels (containing ME or ME-free) was higher than 1, confirming the pseudoplastic flow. Farrow’s constant confirmed the tendency of the system to reduce viscosity due to mechanical stress.

Gels were stored at 4 °C for 6 months. After this period, their rheological properties were re-evaluated. In Carbopol gel, the initially pseudoplastic flow turned into thixotropic with pronounced hysteresis. In Xanthan gel, the area between the curves representing hysteresis increased significantly. Thixotropic systems are advantageous in terms of application and storage. After 6 months of storage, a decrease in the viscosity of both gels was observed, by 29.1% for Carbopol gel and 49.4% for Xanthan gel. The results led to a conclusion in favor of synthetic polymer gels. Natural gels underlie rapid degradation, which was confirmed by the results of rheological measurement.

#### 3.2.3. Texture Analysis

In the development of dermal dosage forms, parameters contributing to the positive acceptance of the drug by the patient are monitored, such as consistency, easy removal of the product from the package, smooth extrusion of the product from the tube, or its good spreadability on the skin or mucous membrane. The clinical effect of semi-solid medicinal products is influenced, *inter alia*, by their adhesion to the skin, satisfactory viscosity, and the drug penetration into the upper layers of the skin. A method of analyzing the texture profile may be used to evaluate the mechanical, rheological, and mucoadhesive properties of dermal semi-solids. For this method, the measuring probe was immersed in the test product for two consecutive cycles.

Due to the texture analysis of the gels, it is possible to compare the texture parameters, such as hardness, adhesiveness, cohesiveness, compressibility, spreadability, and stickiness. In addition, by comparing product texture after a certain storage period, it is possible to comment on its stability and characterize signs of instability.

Hurler et al. [32] reported that the optimal starting position of the probe for measuring Carbopol and Poloxamer gels is exactly below the surface of the gel. The hardness of the sample indicates the force required to overcome the tension arising after the contact of the probe with the surface of the sample [33]. Cohesiveness, adhesiveness, and strength of hydrogels increase linearly with increasing polymer concentration [32].

Kulawik-Pioro et al. [34] presented a scheme of a texture curve analysis, according to which it is possible to quantify the hardness of the product as the maximum force at the first compression, adhesiveness as the area of the negative peak in the first cycle of the measurement, cohesiveness as the ratio of the area of the positive peaks in the second and first cycle, and compressibility as the area of the positive peak in the first cycle.

**Figure 4:**
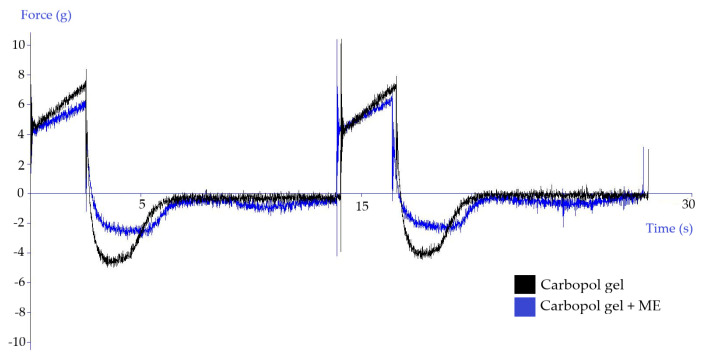
The influence of the reference microemulsion (ME) on the texture of Carbopol gel.

**Figure 5:**
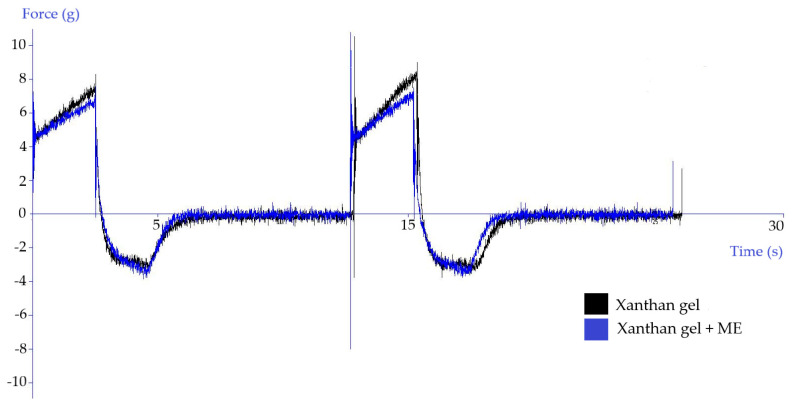
The influence of the reference microemulsion (ME) on the texture of Xanthan gel.

The results of the texture analysis of the gels, depending on the presence of the reference microemulsion (ME), are shown in Figure 4 and Figure 5. The hardness of the Carbopol gel was reduced due to the addition of the microemulsion. The adhesiveness was reduced by almost half. The cohesiveness remained almost unchanged. The compressibility was slightly reduced in the microemulsion-containing gel. In the case of Xanthan gel, the effect of the presence of the microemulsion on the texture properties of the gel was not so pronounced. The hardness and compressibility of the Xanthan gel were slightly reduced. In contrast, the adhesiveness and cohesiveness of the gel remained almost unchanged.

### 3.3. In Vitro Drug Release

**Figure 6:**
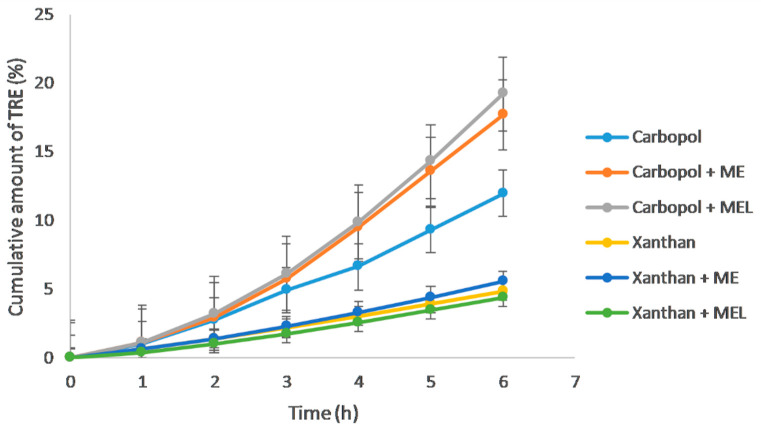
In vitro release of tretinoin from the reference gels, the gels containing the reference microemulsion (ME), or the gels containing the lecithin-containing microemulsion (ME_L_) as drug solubilizers via the synthetic membrane (*n* = 6).

As the in vitro drug release profiles (Figure 6) indicate, a more suitable vehicle for TRE release appears to be Carbopol gel, from which a 2.5-fold higher amount of the drug was released, comparing the reference gels (Carbopol vs. Xanthan gel without previous drug solubilization in any microemulsion) after 6 h. As was mentioned in the Methods (Section 2.5), in the reference gel formulations the drug was solubilized in the mixture of ethanol (96%) and propylene glycol (1:1). Due to the drug solubilization in the reference microemulsion (ME), the cumulative amount of the released drug after 6 h increased by 48%, and after the drug solubilization in lecithin-containing microemulsion (ME_L_) even by 60% in Carbopol gel. In Xanthan gel, the influence of the microemulsions was not so noticeable. The solubilization in ME caused an increase in a cumulative drug amount only by 14%, and on the contrary, solubilization in ME_L_ caused its decrease by 10%. The liberation profiles show that the difference in the drug release occurs after approximately three hours when the drug amount released through the membrane begins to increase significantly compared to the reference Carbopol gel. Considering the synthetic membrane simulates the drug passage through the least permeable upper layer of the skin, the *stratum corneum*, it would mean that the microemulsions act as penetration enhancers, which support the drug transport to the deeper layers of the skin, where the drug is supposed to act. The important question is to what extent the microemulsion promotes the drug absorption up to the blood circulation, which is no longer desirable in the case of the topical treatment of dermal diseases such as acne. This is possible to investigate most simply using an ex vivo permeation test, in which a skin model, whether human, animal, or artificial, is used instead of a synthetic membrane.

Data obtained during the in vitro drug release from the Carbopol and Xanthan gel, when ME was used as the drug solubilizer, were not statistically significant (*p* > 0.05) compared to the reference gels without previous drug solubilization in ME. On the contrary, data obtained for Carbopol and Xanthan gels containing ME_L_ were significantly different (*p* ˂ 0.05).

### 3.4. Ex Vivo Drug Permeation Study

**Figure 7:**
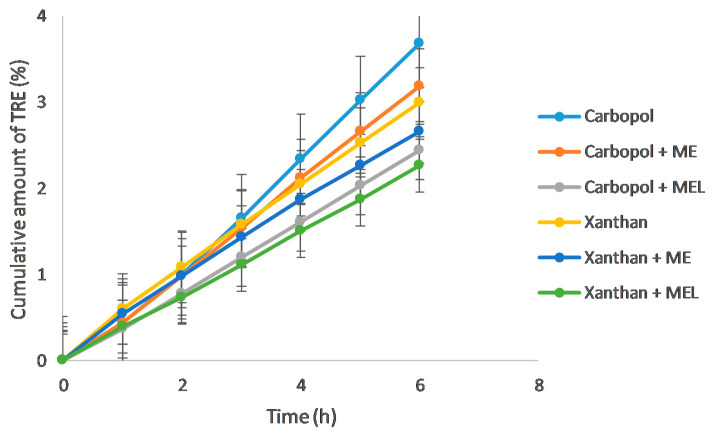
Ex vivo permeation of tretinoin from the reference gels, the gels containing the reference microemulsion (ME), or the gels containing the lecithin-containing microemulsion (ME_L_) as drug solubilizers via pig´s ear skin (*n* = 4).

The results of an ex vivo permeation study through the pig’s ear skin (Figure 7) again confirm the highest released amounts of the drug after six hours precisely from Carbopol compared to Xanthan gel. However, it appears that the solubilization of TRE in microemulsion has rather caused a slowdown in the permeation of the drug through the skin into the dissolution medium, most notably with the lecithin-containing microemulsion ME_L_. After 6 h, the permeated amount of the drug from the gel formulations increased in the following order: Xanthan + ME_L_ > Carbopol + ME_L_ > Xanthan + ME > Xanthan > Carbopol + ME > Carbopol. This action of microemulsion in the dermal application of drugs is advantageous provided that ME_L_ causes increased accumulation of the drug in the skin but prevents its passage into the bloodstream.

Data obtained as the ex vivo permeation profiles were significantly different (at *p* ˂ 0.05) compared to the reference gels (without previous drug solubilization in the microemulsion), regardless of which type of the microemulsion was used.

**Figure 8:**
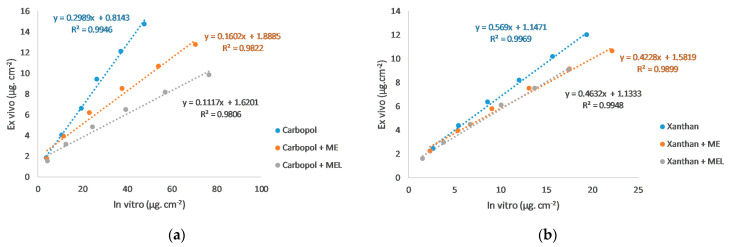
The correlation between the in vitro (synthetic membrane) and the ex vivo (animal skin) drug release: (**a**) Carbopol gel; (**b**) Xanthan gel.

Linear regression was used to correlate the drug release through the synthetic membrane (in vitro) and the animal skin (ex vivo) [35]. The coefficients of determination (R^2^) in Figure 8 demonstrated a high degree of correlation between in vitro and ex vivo data (µg cm^−2^), which means that the ex vivo course of the drug delivery can be predicted based on in vitro data.

The steady-state flux (J_SS_) and permeability coefficient (C_P_) were calculated according to the equations in the paper [19]. The values are reported in Table 4. The enhancement ratio (ER) is expressed as the ratio of the flux from the microemulsion-gel formulation to the flux from the reference gel without the microemulsion (ME or ME_L_). The ER most clearly reflected the influence of the drug solubilization in the microemulsion. As can be seen, ER values belonging to the in vitro drug release data were higher than 1 (or at least close to 1), confirming the positive effect of the microemulsion in the formulation. On the contrary, during the ex vivo permeation studies, both microemulsions caused a slowing down of the drug release (ER ˂ 1).

### 3.5. Drug Release Kinetics

The drug release kinetics from the formulations can be determined utilizing the coefficients of determination (R^2^) characterizing the linear dependence between the certain characteristics of three kinetic models: zero-order, first-order, and Higuchi. Table 5 summarizes the R^2^ values, from which it is obvious that under both conditions, in vitro and ex vivo, the drug release followed the zero-order kinetics (the highest R^2^ values), referring to the constant drug release, and even microemulsion as the drug solubilizer did not change it.

### 3.6. Drug Deposition in Skin

The test has confirmed that microemulsions cause an increase in the accumulation of TRE in the skin, regardless of whether it is Carbopol or Xanthan gel (see Table 6). The reference microemulsion (ME) and lecithin-containing microemulsion (ME_L_) caused a 1.3-fold higher accumulation of TRE in the skin when Carbopol gel was used. The effect of microemulsion action on the amount of the active substance in the skin after the application of Xanthan samples has already varied. ME caused a 1.60-fold increase and ME_L_ slightly lower, with a 1.38-fold increase in the accumulation of the active substance in the skin. As mentioned above, the drug solubilization in the tested microemulsions is advantageous in topical application, provided that ME_L_ causes increased accumulation of the drug in the skin but prevents its passage into the bloodstream. Similar results were noted in the previous study with minoxidil (MXD) [19]. The solubilization of MXD in lecithin-containing microemulsion caused a decrease in the amount of the drug permeated through the skin and, at the same time, an increase in the deposition of the drug in the skin. In summary, both microemulsions used as TRE solubilizers are effective penetration enhancers, and at the same time prevent absorption into the bloodstream.

## 4. Discussion

TRE is a widely used retinoid for the local treatment of acne, psoriasis, skin photo-aging, squamous cell carcinoma of the skin, melasma, follicular keratosis, stretch marks, and skin keratinization disorder [2,5,6]. It is also accepted as a cosmetically beneficial therapy for photo-damaged skin [36]. It normalizes the desquamation of the follicular epithelium, and it regulates the growth and differentiation of epithelial cells, sebum production, and collagen synthesis [5,37]. Its anti-comedogenic and comedolytic activity is mediated by the interaction with cytosol proteins CRABP (cellular retinoic acid binding proteins) and two classes of nuclear retinoid receptors, RAR (retinoic acid receptors) and RXR (retinoid X receptors), which are present in human skin, predominantly in epidermal keratinocytes and dermal fibroblasts [22]. The anti-inflammatory effect is the result of a decrease in the expression of toll-like receptor 2 and a decrease in the release of *Propionibacterium acnes*-induced cytokines [14]. The transdermal absorption and systemic bioavailability of TRE are not desirable, precisely because of its most serious side effect: teratogenicity. The teratogenic effect is induced by the interference of exogenous retinoic acid with its endogenous signaling, which plays a role in embryogenesis. Kriangkrait et al. [38] stated that the exogenous retinoic acid increases the signaling in the mesenchyme underneath the epithelium lining, resulting in induced mesenchymal cell apoptosis.

To reach the target after topical administration, the drug has to diffuse through the corner layer of the skin into the epidermis and dermis [28], thus overcoming a layer of little permeable dead cells to achieve living metabolizing cells [5]. Overcoming the barrier function of the *stratum corneum* can be achieved through different approaches.

The colloidal systems for targeted topical drug delivery such as polymer and solid lipid nanoparticles, nanostructured lipid carriers, liposomes, liposome-similar vesicles, nanoemulsions, and microemulsions are strategic means to improve the efficacy, safety, and tolerability of TRE [18]. They provide drug stabilization and improved drug delivery, thus increasing the effectiveness of skin disease treatment. TRE encapsulation in nanocarriers leads also to a reduction in photo-degradation [17], e.g., Gollnick and Krautheim [39] stated that the incorporation of TRE in microsponges and propolymers increases the tolerability significantly. Rahman et al. [8] studied TRE-loaded liposomal formulation, and in the other paper [4] proniosomes, in which TRE was encapsulated to overcome solubility difficulties and skin irritancy. After the application of niosomal gel, only slight erythema (score 0.143 ± 0.377) was observed. Sabouri et al. [40] studied a nanoemulsion (composed of caprylic/capric triglyceride, Tween 80, and deionized water) as a topical carrier of TRE. However, as is generally known, the preparation of nanoemulsion is an energy-demanding process. In the mentioned study, high-pressure homogenization was used for the transformation from the emulsion to the nanoemulsion. In contrast, microemulsions are formed spontaneously. The microemulsion system is defined as a spontaneously forming, thermodynamically stable, transparent, macroscopically homogeneous, optically isotropic colloidal dispersion (type w/o or o/w or bicontinuous), stabilized at the interface by surfactants and co-surfactants [41]. However, it is necessary to precisely weigh and maintain the proportions of the individual components. Simple preparation and low cost make microemulsion a perfect tool for improving the stability and bioavailability of TRE. In addition, Mortazavi et al. [6] compared the in vitro liberation of TRE from a microemulsion system, a gel, and a cream. Of course, the microemulsion as the least viscous system provided the highest liberation profiles. However, the direct application of solution-like microemulsion on the face is not very effective or tolerable. The authors consider the incorporation of TRE in the microemulsions to be advantageous for three reasons: (i) microemulsions can increase the drug release profile and drug bioavailability; (ii) it might minimize TRE local adverse reactions (such as erythema, flaking, and skin irritation); and (iii) it protects TRE from photo-degradation. In our study, we decided to use the microemulsion only as a drug solubilizer with subsequent incorporation into a gel. The reference microemulsion (ME) was composed of an aqueous phase (distilled water), an oily phase (isopropyl myristate), a surfactant (Tween 80), and a co-surfactant (isopropyl alcohol). In the microemulsion ME_L_, the solution of lecithin (0.5%; *w*/*w*) in isopropyl myristate was added.

Generally, penetration, permeation, and therefore finally TRE bioavailability might be improved by solubilization in the microemulsion vehicle due to the interaction of several mechanisms. The small diameter of the oily droplets (inner phase of o/w microemulsion) ensures the solubilization of poorly water-soluble drugs. Further, surfactants and co-surfactants can reduce the barrier function of the *stratum corneum* acting as so-called penetration enhancers [41], e.g., isopropyl alcohol present in the microemulsions interacts with the polar groups of lipid components of the *stratum corneum* [42]. The further mechanism is based on the increased thermodynamic activity of the drug due to the microemulsion, thereby increasing its distribution into the skin [43].

The prepared microemulsions, which did not differ from each other in their organoleptic characteristics (light yellow coloration, transparent, light alcohol odor), were further evaluated in terms of their physicochemical properties. Information about inner droplet size, polydispersity index, density, viscosity, surface tension, conductivity, and pH are noticed in the previous study [19]. The influence of the presence of lecithin in the system on the physical parameters was minimal. Due to low viscosity, the microemulsions appeared to be unsuitable vehicles for topical or transdermal drug delivery alone. They are difficult to spread on the face, and the high content of Tween 80 can be irritating to the skin. Therefore, TRE solubilized in the microemulsion was further formulated to hydrogel, forming three-dimensional networks of hydrophilic polymer chains [31].

The surfactant lecithin has beneficial effects on drug penetration through the skin, as evidenced by several studies investigating its effect either in a mixture with terpenes or as part of vesicles [44]. Lecithin is a convenient choice of surfactant for a microemulsion formation, because of its natural origin, biocompatibility, and non-toxicity. Even at high concentrations, it is skin non-irritating, which is the main benefit compared to synthetic surfactants. Generally, phospholipids are recognized as chemical penetration enhancers since the contact of phospholipids with the skin might cause skin hydration, and subsequently an increase in the drug concentration in the tissue. Lecithin-based microemulsions have a natural affinity to the cell membrane, causing an enhancement in the dermal and transdermal absorption of several drugs [45].

The main disadvantage is that lecithin is too lipophilic in water-oil systems to spontaneously form the zero mean curvature amphiphile layers needed for microemulsion formation [46]. Precisely because the creation of a spontaneously formed microemulsion containing lecithin is difficult and requires a precise choice of the additional surfactant and co-surfactant, soybean or egg yolk lecithin is rather used in coarse emulsions and nanoemulsions, especially in preparations intended for parenteral nutrition [47]. The phospholipid-based microemulsion was tested only as a parenterally administered TRE carrier in the treatment of promyelocytic leukemia, and was shown to be a suitable alternative to the oral treatment [48].

By adding lecithin to the microemulsion, it was intended to only slightly improve the penetration into the *stratum corneum*, so that the drug remained as much as possible in the skin (*dermis*) where it should act therapeutically, with a maximum limitation of systemic action. Despite the fact that the effect of lecithin in microemulsion appeared to be small according to the result of the drug deposition study, the microemulsion itself significantly supported its retention in the skin. It appears that the amount of lecithin addition in the microemulsion was too low to cause more significant differences. On the contrary, in the ex vivo permeation test, the microemulsion containing lecithin in both cases (in Xanthan and also Carbopol gel) caused a reduction in the drug concentration diffused through the skin into the dissolution medium simulating blood circulation, which is a desirable effect in the therapy of acne vulgaris, as well as for all diseases treated topically.

TRE is practically insoluble in water, soluble in DMSO, and slightly soluble in polyethylene glycol 400, octanol, and ethanol [1]. The poor solubility of TRE in water limits its incorporation into aqueous vehicles. Therefore, the use of relatively irritating auxiliary solvents such as ethanol and propylene glycol in commercial products is required [2]. In addition to TRE, the same problem also exists with many other lipophilic active substances primarily intended for topical or transdermal administration. The development of their topical dosage forms is currently focusing on so-called “lipophilic formulations” to increase their bioavailability. These formulations (liposomes, nanoparticles, self-emulsifying systems, etc.) use good drug solubility in fats [29]. The Draize test on rabbit skin confirmed the lower irritant potential of such a formulation compared to the commercial preparation [49]. Therefore, it is assumed that due to the encapsulation of TRE in such drug carriers or the tested microemulsions, its acidic functional groups are in reduced contact with the *stratum corneum*, which is thought to trigger erythematous episodes. Ghate et al. [50] compared a commercially available TRE gel and formulation with TRE encapsulated in nanostructured lipid particles (NLCs). The results showed that using the formulation with NLCs, a lower irritant potential of TRE, a higher content in the skin, and a prolonged release from the gel formulation were achieved. Furthermore, the crystal structure of NLCs results in light scattering and thus is good protection for photo-labile drugs.

Bakshi et al. [51] evaluated the skin irritation of a nanoemulsion with a similar composition to our microemulsions (IPA, IPM, Tween 80, Span 20, demineralized water) by an MTT assay on Epiderm^TH^ tissue and by a cytokine analysis on 3D cell culture. The results of both tests predicted the non-irritating nature of the formulation. Gupta and Moulik [52] summarized biocompatible microemulsions composed of prospective components. One of them had a similar composition to ours with the difference in the used co-surfactant (propylene glycol instead of IPA). Based on the data, we assume the biocompatibility of the microemulsions used.

Through the texture and rheological characteristics of the prepared semi-solid formulations, it is possible to compare the simplicity of their topical application (spreadability) and stability. 

There are several methods to simulate the penetration of the drug through the skin. In the study, the drug release was investigated by passing through the synthetic membrane and the skin from the pig’s ear. The synthetic membrane is generally considered to be more permeable than biological materials, and is not suitable for the investigation of penetration enhancer action [53]. However, it can easily depict the *stratum corneum*, that is, the upper least-permeable layer of the skin, simulating the process of liberation (drug release from the gel to the interface with the horn layer) and penetration (the drug delivery into the horn layer). SpectraPor^®^ is not a routinely used or validated membrane such as Strat-M or other equivalents for in vitro drug release studies. However, it was used in several in vitro permeation studies, according to which we proceeded [54,55].

Through the in vitro drug release test, the information on the rate and the amount of TRE that was released and subsequently penetrated the dissolution medium was obtained. During in vitro permeation studies, a phosphate buffer solution (PBS) was first used as a dissolution medium, but it was found that due to the TRE lipophilic character, no drug amount was diffused through the membrane into the PBS. To support the penetration of the drug through the membrane, we had to add ethanol into the dissolution medium, in which the drug is slightly soluble. The addition of alcohol to the dissolution medium is a common procedure during permeation studies, improving the diffusion of a poorly soluble drug into the dissolution medium. Bagatin et al. [5] used a 10% aqueous-methanol solution, Bavarsad et al. [37] used a methanol and phosphate buffer (1:2), Raza et al. [2] used an ethanolic solution, and Mortazavi et al. [6] used ethanol (96%) and a phosphate buffer at a ratio of 1:2 as dissolution media. Moghimi et al. [56] have also reported that permeation of TRE and isoTRE in the absence of enhancers through full-thickness rat skin was rather low, and they were not able to detect the retinoids in the receptor phase even by the HPLC method. Although the dissolution medium is not physiological, it is possible to compare the changes in the drug release caused by the composition of the formulation under the same conditions, i.e., to evaluate the influence of the excipients in the vehicle.

The amount of samples applied on the membrane during the in vitro/ex vivo drug release was experimentally defaulted so that it covered the surface of the synthetic membrane or the skin in a thin layer, thus imitating a dermal application. In addition, the summary of the product characteristics of commercially available gel with the content of TRE and clindamycin recommends applying a layer of a pea size to the affected area, which corresponds to an amount of approximately 0.6 g. The amount of TRE in the gel was 40 times higher than in the mentioned commercial product. At a lower concentration, the amount diffused through the membrane could not be spectrophotometrically determined.

After the replacement of the synthetic membrane with animal skin, an ex vivo model was obtained and allowed to determine the drug amount not only released from the dosage form and having penetrated the upper layer of the skin, but also the amount that had permeated into the deeper layers of the skin and subsequently “reached” systemic circulation.

Barbero and Frasch [57] stated that, based on morphological and functional data, pig skin appears to be the most similar to human skin. This is mainly due to the similarity in biochemical properties, follicle density, and thickness of the *stratum corneum* [24]. Even the low value of the intraspecific coefficient of pig skin (21%) defines it as an excellent substitute for human skin with a coefficient of variation of up to 35%. The skin pieces used before in the ex vivo drug permeation study were macerated in propylene glycol at 4 °C. Propylene glycol appears to be a suitable extraction agent not only because of the good solubility of TRE, but also because of its miscibility with the aqueous-ethanolic solution, which was used for dilution of the samples. Based on the results of the drug solubility, in vitro liberation tests, and ex vivo permeation tests, we assumed that the greatest amount of TRE would be retained in the skin to which a sample of Carbopol gel with drug solubilized in lecithin-containing microemulsion was applied. Therefore, the result of the determination of drug deposition in the skin, which demonstrates the approximately same amount of TRE detected in the skin regardless of whether the microemulsion contained lecithin, was surprising. In summary, both tested microemulsions appear to be suitable drug solubilizers and penetration enhancer systems delivering the drug to the dermis, where TRE should act. Many other studies monitored the drug release through the layers of the skin during ex vivo permeation studies, e.g., through the *stratum corneum* separated from the skin or after the separation of the epidermis and dermis layers. To simplify the experiment, we did not separate the layers of the skin; however, this type of investigation could also bring instructive results.

There are justified concerns about porcine ear skin use in ex vivo penetration experiments, especially due to the preservation of integrity and skin condition during cold or frozen storage. An evaluation of skin integrity by TEWL is recommended, although some authors consider this method less appropriate. Keck et al. [58] reported that high TEWL values and high skin hydration are not representative enough to accurately identify the skin barrier properties because *rigor mortis* causes high TEWL values and high skin hydration but reduces skin permeability.

TRE is a drug with a short half-life of 0.5–2 h. Therefore, we performed liberation studies only up to the 6th hour. In addition, it is recommended to apply the TRE dermal products in the evening to avoid degradation by daylight, since they are photo-labile. In the case of stable drugs, a statistical calculation of the expected release of the drug would also come into consideration, as was completed by Bavarsad et al. [37] after an 8 h liberation study.

Xanthan gum is an anionic polysaccharide biopolymer derived from microorganisms [59]. It is produced by the fermentation activity of the Gram-negative bacteria *Xanthomonas campestris* under aerobic conditions in a sterile environment [60]. Compared to synthetically produced Carbopol, it has the advantage of an organic origin. In medical cosmetics, there is a growing trend to replace synthetic excipients with natural ones. This was also one of the goals of our investigation. Unfortunately, as it was evidenced, TRE was released more slowly and penetrated the skin to a lesser extent from Xanthan gel as a vehicle compared to Carbopol. It would be suitable to investigate Xanthan gels of lower viscosity (lower concentration of the gelling agent) which should be in favor of the drug release.

## 5. Conclusions

The results obtained are useful in practice in the formulation of a dermal TRE gel. Based on the results, Carbopol gel might be considered more suitable among the tested vehicles, and lecithin-containing ME_L_ might be recommended to solubilize the active substance. Although lecithin in ME_L_ did not cause a sharp increase in the deposition amount of the active substance in the skin, the results of the ex vivo tests indicate that it could prevent the absorption of the active substance into the bloodstream, and thus limit undesirable effects resulting from systemic action.

## Figures and Tables

**Table 1:** The composition of the microemulsions and the gels.

	ME (*w*/*w*)	ME_L_ (*w*/*w*)	Carbopol Gel (*w*/*w*)	Xanthan Gel (*w*/*w*)
Tween 80	26.50	26.50	-	-
Isopropyl alcohol	26.50	26.50	-	-
Isopropyl myristate	16.00	15.25	-	-
Lecithin ^1^	-	0.75	-	-
Carbopol	-	-	1.00	-
Xanthan gum	-	-	-	1.50
NaOH ^2^	-	-	4.00	-
Distilled water	31.00	31.00	95.00	98.50

^1^ 0.5% solution in isopropyl myristate; *w*/*w*. ^2^ 10% aqueous solution; *w*/*w*.

**Table 2:** Solubility of tretinoin (TRE) in hydrophilic components of the microemulsion or the microemulsions themselves.

Solvent	A	Dilution	TRE Concentration (µg·mL^−1^)
Isopropyl alcohol	0.107 ± 0.012	1: 2500	2400.66 ± 248.63
Tween 80	0.071 ± 0.010	1: 1000	662.25 ± 82.78
Water	0.194 ± 0.010	1: 50	84.02 ± 4.14
ME	0.857 ± 0.006	1: 500	3584.43 ± 26.61
ME_L_	0.848 ± 0.005	1: 500	3547.19 ± 21.24

**Table 3:** Farrow’s constants (n).

Gel	N	
	+	−
Carbopol	1.325	1.140
Carbopol + ME	1.208	1.246
Xanthan	1.138	1.186
Xanthan + ME	1.202	1.154

^(+)^ up-curve; ^(−)^ down-curve.

**Table 4:** Steady-state flux (J_SS_), permeability coefficient (C_P_), and enhancement ratio (ER) comparing the microemulsion gel formulation to the reference gel under the in vitro and ex vivo conditions.

	In Vitro			Ex Vivo		
	J_SS_ (µg cm^−2^ h^−1^)	ER	C_P_ (10^−4^)	J_SS_ (µg cm^−2^ h^−1^)	ER	C_P_ (10^−4^)
Carbopol	9.15	-	7.63	2.70	-	2.25
Carbopol + ME	14.93	1.63	12.44	2.21	0.82	1.84
Carbopol + ME_L_	16.04	1.75	13.37	1.66	0.61	1.38
Xanthan	3.49	-	2.91	1.91	-	1.59
Xanthan + ME	4.20	1.20	3.50	1.67	0.87	1.39
Xanthan + ME_L_	3.43	0.98	2.86	1.52	0.80	1.27

**Table 5:** The coefficients of determination (R^2^) for zero-order kinetics, first-order kinetics, and the Higuchi model.

	Carbopol	Carbopol + ME	Carbopol + ME_L_	Xanthan	Xanthan + ME	Xanthan + ME_L_
In Vitro	R^2^	R^2^	R^2^	R^2^	R^2^	R^2^
Zero-order	0.9922	0.9839	0.9798	0.9974	0.995	0.9934
First-order	0.9245	0.9429	0.9439	0.9488	0.9454	0.9382
Higuchi	0.9587	0.9383	0.931	0.9698	0.9624	0.9583
Ex Vivo						
Zero-order	0.9987	0.9998	1.0000	0.9999	0.9989	0.9997
First-order	0.9357	0.9232	0.9318	0.9458	0.9399	0.9458
Higuchi	0.9758	0.9858	0.9839	0.9858	0.9902	0.9813

**Table 6:** The concentration c (µg mL^−1^) of tretinoin determined in the skin macerate after the ex vivo permeation study of the tested formulations.

	A	Ā	S.D.	c (µg·mL^−1^)	$\bar{c}$ (µg·mL^−1^)	S.D.
Carbopol	0.204	0.227	0.023	1.763	1.954	0.190
	0.250			2.144		
	0.227			1.954		
Carbopol + ME	0.255	0.287	0.031	2.185	2.448	0.253
	0.316			2.690		
	0.289			2.467		
Carbopol + ME_L_	0.253	0.286	0.046	2.169	2.442	0.377
	0.338			2.873		
	0.267			2.285		
Xanthan	0.101	0.131	0.043	0.911	1.159	0.354
	0.112			1.002		
	0.180			1.565		
Xanthan + ME	0.226	0.217	0.009	1.945	1.871	0.075
	0.217			0.871		
	0.208			0.796		
Xanthan + ME_L_	0.191	0.187	0.007	0.656	1.625	0.060
	0.179			0.556		
	0.192			0.664		

## Data Availability

Not applicable.

## References

[1] PubChem Tretinoin. https://pubchem.ncbi.nlm.nih.gov/compound/444795.

[2] Raza K., Singh B., Lohan S., Sharma G., Negi P., Yachha Y., Katare O.P. (2013). Nano-lipoidal carriers of tretinoin with enhanced percutaneous absorption, photostability, biocompatibility and anti-psoriatic activity. Int. J. Pharm..

[3] Yoham A.L., Casadesus D. (2022). Tretinoin. StatPearls.

[4] Rahman S.A., Abdelmalak N.S., Badawi A., Elbayoumy T., Sabry N., Ramly A.E. (2015). Formulation of tretinoin-loaded topical proniosomes for treatment of acne: In-vitro characterization, skin irritation test and comparative clinical study. Drug Deliv..

[5] Bagatin E., Wagemaker T.A.L., Aguiar Júnior N.d.R., Gianeti M.D., Gonçalves E.M.B., Campos P.M.B.G.M. (2015). Tretinoin-based formulations—influence of concentration and vehicles on skin penetration. Braz. J. Pharm. Sci..

[6] Mortazavi S.A., Pishrochi S. (2013). Formulation and In-vitro Evaluation of Tretinoin Microemulsion as a Potential Carrier for Dermal Drug Delivery. Iran. J. Pharm. Res..

[7] Darlenski R., Surber C., Fluhr J.W. (2010). Topical retinoids in the management of photodamaged skin: From theory to evidence-based practical approach. Br. J. Dermatol..

[8] Rahman S.A., Abdelmalak N.S., Badawi A., Elbayoumy T., Sabry N., El Ramly A. (2016). Tretinoin-loaded liposomal formulations: From lab to comparative clinical study in acne patients. Drug Deliv..

[9] Loureiro K.D., Kao K.K., Jones K.L., Alvarado S., Chavez C., Dick L., Felix R., Johnson D., Chambers C.D. (2005). Minor malformations characteristic of the retinoic acid embryopathy and other birth outcomes in children of women exposed to topical tretinoin during early pregnancy. Am. J. Med. Genet. A.

[10] Szymański Ł., Skopek R., Palusińska M., Schenk T., Stengel S., Lewicki S., Kraj L., Kamiński P., Zelent A. (2020). Retinoic Acid and Its Derivatives in Skin. Cells.

[11] Melhorn A. (2017). Tretinoin. Hautarzt.

[12] Shannon J.F. (2020). Why do humans get acne? A hypothesis. Med. Hypotheses.

[13] Schmidt N., Gans E.H. (2011). Tretinoin: A Review of Its Anti-inflammatory Properties in the Treatment of Acne. J. Clin. Aesthetic Dermatol..

[14] Ourique A.F., Melero A., da Silva C.D.B., Schaefer U.F., Pohlmann A.R., Guterres S.S., Lehr C.-M., Kostka K.-H., Beck R.C.R. (2011). Improved photostability and reduced skin permeation of tretinoin: Development of a semisolid nanomedicine. Eur. J. Pharm. Biopharm..

[15] Ioele G., Cione E., Risoli A., Genchi G., Ragno G. (2005). Accelerated photostability study of tretinoin and isotretinoin in liposome formulations. Int. J. Pharm..

[16] Rosso J.D., Harper J., Pillai R., Moore R. (2013). Tretinoin Photostability. J. Clin. Aesthetic Dermatol..

[17] Morales J.O., Valdés K., Morales J., Oyarzun-Ampuero F. (2015). Lipid nanoparticles for the topical delivery of retinoids and derivatives. Nanomedicine.

[18] Latter G., Grice J.E., Mohammed Y., Roberts M.S., Benson H.A.E. (2019). Targeted topical delivery of retinoids in the management of acne vulgaris: Current formulations and novel delivery systems. Pharmaceutics.

[19] Špaglová M., Cuchorova M., Čierna M., Bauerova K., Poništ S. (2021). Microemulsions as Solubilizers and Penetration Enhancers for Minoxidil Release from Gels. Gels.

[20] Xie M., Liu X., Wang S. (2022). Degradation of methylene blue through Fenton-like reaction catalyzed by MoS2-doped sodium alginate/Fe hydrogel. Colloids Surf. B Biointerfaces.

[21] Arora R., Aggarwal G., Harikumar S.L., Kaur K. (2014). Nanoemulsion Based Hydrogel for Enhanced Transdermal Delivery of Ketoprofen. Adv. Pharm..

[22] Brisaert M., Plaizier-Vercammen J.A. (2007). Investigation on the photostability of tretinoin in creams. Int. J. Pharm..

[23] Tashtoush B.M., Jacobson E.L., Jacobson M.K. (2008). UVA is the major contributor to the photodegradation of tretinoin and isotretinoin: Implication for development of improved pharmaceutical formulations. Int. J. Pharm..

[24] Ascenso A., Vultos F., Ferrinho D., Salgado A., Filho S.G., Ferrari V., Simões S., Marques H.C. (2011). Effect of tretinoin inclusion in dimethyl-beta-cyclodextrins on release rate from a hydrogel formulation. J. Incl. Phenom. Macrocycl. Chem..

[25] Xu H., Zhang F., Wang M., Lv H., Yu D.-G., Liu X., Shen H. (2022). Electrospun hierarchical structural films for effective wound healing. Biomater. Adv..

[26] Scomoroscenco C., Teodorescu M., Raducan A., Stan M., Voicu S.N., Trica B., Ninciuleanu C.M., Nistor C.L., Mihaescu C.I., Petcu C. (2021). Novel Gel Microemulsion as Topical Drug Delivery System for Curcumin in Dermatocosmetics. Pharmaceutics.

[27] Dash S., Murthy P.N., Nath L., Chowdhury P. (2010). Kinetic Modeling on Drug Release from Controlled Drug Delivery Systems. Acta Pol. Pharm..

[28] Sezer A.D. (2014). Application of Nanotechnology in Drug Delivery.

[29] Franc A., Smilková V., Rabišková M., Vetchý D., Kratochvíl B. (2012). Lipofilní formulace pro zvýšení biodostupnosti těžce rozpustných léčivých látek. Chem. Listy.

[30] Gibaud T., Divoux T., Manneville S. (2020). Nonlinear mechanics of colloidal gels: Creep, fatigue and shear-induced yielding. Statistical and Nonlinear Physics.

[31] El-Leithy E.S., Shaker D.S., Ghorab M.K., Abdel-Rashid R.S. (2010). Evaluation of Mucoadhesive Hydrogels Loaded with Diclofenac Sodium–Chitosan Microspheres for Rectal Administration. AAPS PharmSciTech.

[32] Hurler J., Engesland A., Poorahmary Kermany B., Škalko-Basnet N. (2012). Improved texture analysis for hydrogel characterization: Gel cohesiveness, adhesiveness, and hardness. J. Appl. Polym. Sci..

[33] das Neves J., Bahia M.F. (2006). Gels as vaginal drug delivery systems. Int. J. Pharm..

[34] Kulawik-Pióro A., Potykanowicz A. (2016). Determining the quality of hydrophobic barrier creams by rheological measurements, sensory analysis, pH determination and permeation time measurements. Chemom. Intell. Lab. Syst..

[35] Blakney A.K., Little A.B., Jiang Y., Woodrow K.A. (2017). In vitro–ex vivo correlations between a cell-laden hydrogel and mucosal tissue for screening composite delivery systems. Drug Deliv..

[36] Honeybrook A., Bernstein E. (2020). Oral isotretinoin and photoaging: A review. J. Cosmet. Dermatol..

[37] Bavarsad N., Akhgari A., Seifmanesh S., Salimi A., Rezaie A. (2016). Statistical optimization of tretinoin-loaded penetration-enhancer vesicles (PEV) for topical delivery. Daru J. Fac. Pharm. Tehran Univ. Med. Sci..

[38] Kriangkrai R., Chareonvit S., Iseki S., Limwongse V. (2017). Pretreatment Effect of Folic Acid on 13-Cis-RA-Induced Cellular Damage of Developing Midfacial Processes in Cultured Rat Embryos. Open Dent. J..

[39] Gollnick H.P.M., Krautheim A. (2003). Topical treatment in acne: Current status and future aspects. Dermatol. Basel Switz..

[40] Sabouri M., Samadi A., Ahmad Nasrollahi S., Farboud E.S., Mirrahimi B., Hassanzadeh H., Nassiri Kashani M., Dinarvand R., Firooz A. (2018). Tretinoin Loaded Nanoemulsion for Acne Vulgaris: Fabrication, Physicochemical and Clinical Efficacy Assessments. Skin Pharmacol. Physiol..

[41] Chen L., Tan F., Wang J., Liu F. (2012). Microemulsion: A novel transdermal delivery system to facilitate skin penetration of indomethacin. Pharmazie.

[42] Güngör S., Bergişadi N. (2004). Effect of penetration enhancers on in vitro percutaneous penetration of nimesulide through rat skin. Pharmazie.

[43] Baroli B., López-Quintela M.A., Delgado-Charro M.B., Fadda A.M., Blanco-Méndez J. (2000). Microemulsions for topical delivery of 8-methoxsalen. J. Control. Release.

[44] Herman A., Herman A.P. (2015). Essential oils and their constituents as skin penetration enhancer for transdermal drug delivery: A review. J. Pharm. Pharmacol..

[45] Gutierrez-Mendez N., Chávez D., Leal-Ramos M. (2022). Lecithins: A comprehensive review of their properties and their use in formulating microemulsions. J. Food Biochem..

[46] Shinoda K., Araki M., Sadaghiani A., Khan A., Lindman B. Lecithin-Based Microemulsions: Phase Behavior and Microstructure. https://pubs.acs.org/doi/pdf/10.1021/j100155a091.

[47] Schuh R., Bruxel F., Teixeira H. (2014). Physicochemical properties of lecithin-based nanoemulsions obtained by spontaneous emulsification or high-pressure homogenization. Quím. Nova.

[48] Hwang S.R., Lim S.-J., Park J.-S., Kim C.-K. (2004). Phospholipid-based microemulsion formulation of all-trans-retinoic acid for parenteral administration. Int. J. Pharm..

[49] Shah K.A., Date A.A., Joshi M.D., Patravale V.B. (2007). Solid lipid nanoparticles (SLN) of tretinoin: Potential in topical delivery. Int. J. Pharm..

[50] Ghate V.M., Lewis S.A., Prabhu P., Dubey A., Patel N. (2016). Nanostructured lipid carriers for the topical delivery of tretinoin. Eur. J. Pharm. Biopharm..

[51] Bakshi P., Jiang Y., Nakata T., Akaki J., Matsuoka N., Banga A. (2018). Formulation Development and Characterization of Nanoemulsion-Based Formulation for Topical Delivery of Heparinoid. J. Pharm. Sci..

[52] Gupta S., Moulik S. (2008). Biocompatible Microemulsions and Their Prospective Uses in Drug Delivery. J. Pharm. Sci..

[53] Berkó S., Balázs B., Sütő B., Eros G., Gál B., Sztojkov-Ivanov A., Soica C., Szabó-Révész P., Kemény L., Zupko I. (2014). Monitoring of skin penetration and absorption with a new in vivo experimental model. Farmacia.

[54] Koutsoulas C., Pippa N., Demetzos C., Zabka M. (2014). Preparation of Liposomal Nanoparticles Incorporating Terbinafine In Vitro Drug Release Studies. J. Nanosci. Nanotechnol..

[55] Koutsoulas C., Suleiman E., Wagner A., Zabka M. (2014). Comparative study between synthetic and phospholipids of natural origin: Effect of phospholipid selection on the behavior of a topical liposomal dosage form incorporating terbinafine. J. Liposome Res..

[56] Moghimi H., Zarghi A., Noorani N. (2003). Stereoselective Permeation of Tretinoin and Isotretinoin through Enhancer-Treated Rat Skin.I. Effect of Ethanol and Sodium Dodecyl Sulfate. Iran. J. Pharm. Res..

[57] Barbero A.M., Frasch H.F. (2009). Pig and guinea pig skin as surrogates for human in vitro penetration studies: A quantitative review. Toxicol. In Vitro.

[58] Keck C.M., Abdelkader A., Pelikh O., Wiemann S., Kaushik V., Specht D., Eckert R.W., Alnemari R.M., Dietrich H., Brüßler J. (2022). Assessing the Dermal Penetration Efficacy of Chemical Compounds with the Ex-Vivo Porcine Ear Model. Pharmaceutics.

[59] Mikušová V., Ferková J., Žigrayová D., Krchňák D., Mikuš P. (2022). Comparative Study of Polysaccharide-Based Hydrogels: Rheological and Texture Properties and Ibuprofen Release. Gels.

[60] Patel J., Moorthy N.S.H.N., Maiti S., Bera H., Hossain C.M., Saha S. (2021). Chapter 11—Xanthan-based nanomaterials for drug delivery applications. Biopolymer-Based Nanomaterials in Drug Delivery and Biomedical Applications.

